# 

**Published:** 1987-05

**Authors:** 


**Contents**

**33 Abdominal symptoms in general practice, a case control study**
Paul R. Bond
**35 Phenytoin (Epanutin) associated Hodgkin's Disease**
T. K. Dahneshmend and J. D. Davies
**37 The dangers of Cassava (Tapioca) consumption**
Michael J. Hall
**40 Gastric carcinoid presenting with haematemesis**
C. F. M. Weston, D. A. Macdonald and A. Rikabi
**41 Oesophageal pain and catecholamine mediated ecg changes mimicking myocardial ischaemia**
Robert F. Logan and John E. Sanderson
**42 Academic practice or primitive physic**
D. G. Craig
**43 Obituary—Dr Terence Steen**
**44 Putting the boot in**
J. L. Burton
**46 From our Correspondents**
**49 From our Foreign Correspondent**
**51 South West Urologists Club, Abstracts of meeting at Taunton**
**54 Book Reviews**
**56 On gardening**
T. F. Hewer

